# Contrast extravasation mimicking intracerebral and intraventricular hemorrhage after intravenous thrombolytic treatment of ischemic stroke: a case report

**DOI:** 10.1186/s12883-024-03618-y

**Published:** 2024-04-19

**Authors:** Jiuning Tang, Xinhai Zhang, Jinhui Yu, Zhi Liu, Huaqiang Ding

**Affiliations:** Department of Neurosurgery, People’s Hospital of Yubei District of Chongqing City, Chongqing, China

**Keywords:** Intravenous thrombolytics, Contrast media, Intracranial hemorrhage, Computed tomography

## Abstract

**Background:**

Although contrast extravasation on follow-up head computed tomography (CT) is frequently visualized after endovascular treatment, this phenomenon is rare after intravenous thrombolytic treatment in patients with acute ischemic stroke (AIS). Here, we report a case of contrast extravasation mimicking intracerebral hemorrhage (ICH) with intraventricular extension after intravenous thrombolytic treatment and computed tomography angiography (CTA).

**Case presentation:**

A 52-year-old man presented with right-sided hemiparesis and hypoesthesia. Initial non-contrast head CT was negative for intracranial hemorrhage and acute ischemic changes. He received intravenous treatment with tenecteplase 3.8 h after the onset of stroke. CTA of the head and neck was performed at 4.3 h after stroke onset. It showed no stenosis or occlusion of the carotid and major intracranial arteries. At about 1.5 h after CTA, the right-sided hemiparesis deteriorated, accompanied by drowsiness, aphasia, and urinary incontinence. Immediate head CT showed hyperdense lesions with mild space-occupying effect in the left basal ganglia and both lateral ventricles. The hyperdense lesions were reduced in size on follow-up CT after 5 h. Two days later, CT showed that the hyperdense lesions in the lateral ventricles almost completely disappeared and only a small amount remained in the infarcted area.

**Conclusions:**

Contrast extravasation into the brain tissue and lateral ventricles, mimicking ICH with intraventricular extension, could occur after intravenous thrombolytic treatment and CTA in a patient with AIS, which might lead to misdiagnosis and wrong treatment of the patient. The rapid resolution of intracranial hyperdense lesions is key to differentiate contrast extravasation from ICH on serial non-enhanced CT.

## Background

Intravenous treatment (IVT) with recombinant tissue-plasminogen activator is recommended for patients with acute ischemic stroke (AIS) who may be treated within 4.5 h of symptom onset [1]. However, it increases the risk of hemorrhagic transformation (HT) and can even lead to fatal intracranial hemorrhage [2]. A follow-up computed tomography (CT) after intravenous thrombolytic treatment is often performed to rule out intracerebral hemorrhage (ICH) before the initiation of antithrombotic agents. However, it is difficult to distinguish ICH from contrast extravasation on conventional head CT due to the similar radiologic appearance of both pathologies. Recently, we encountered a rare case of contrast extravasation mimicking ICH with intraventricular extension after IVT with tenecteplase and computed tomography angiography (CTA). We are trying to interpret the unusual phenomenon through review of the previous literature.

## Case presentation

**Fig. 1:**
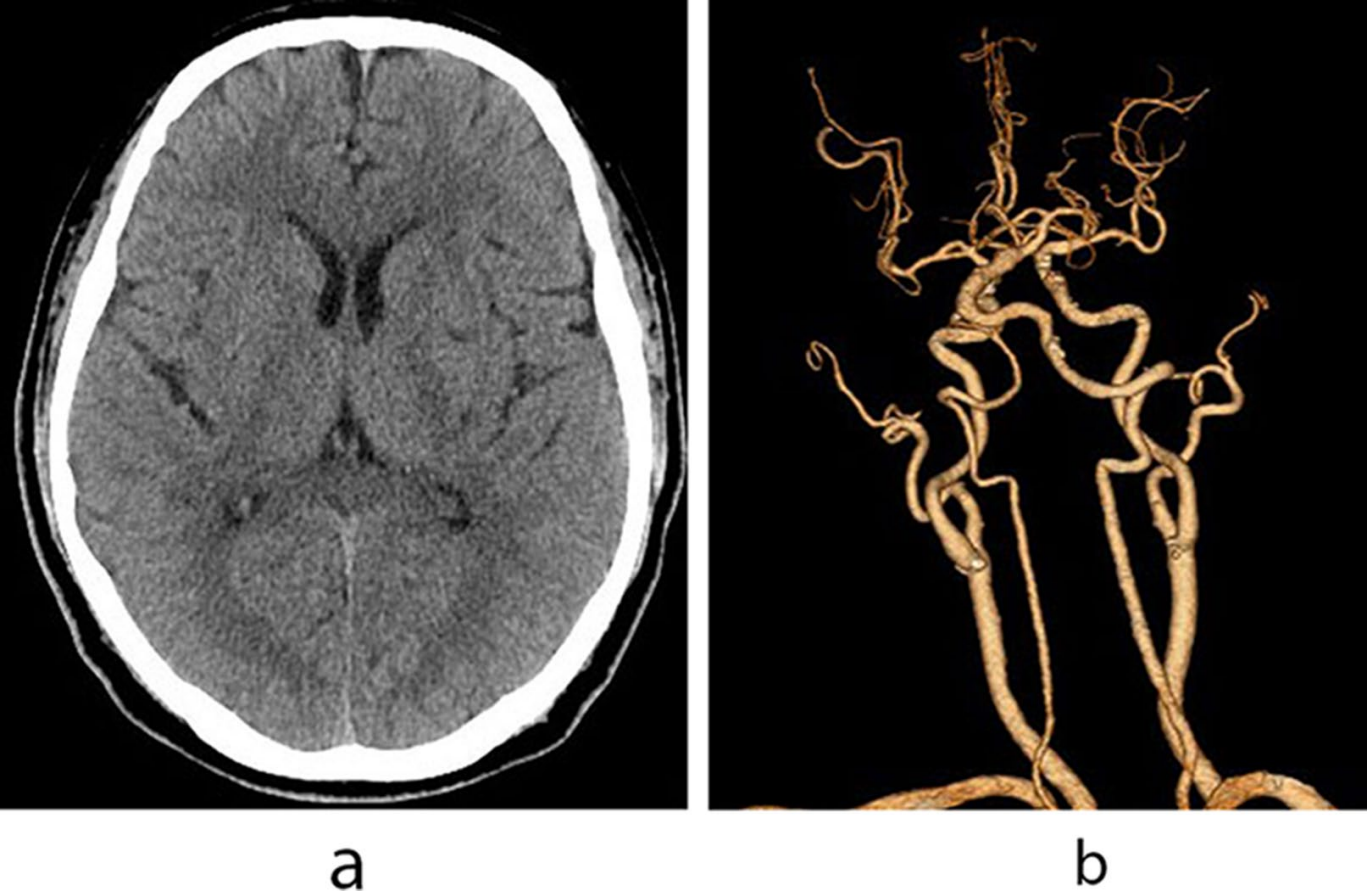
(**A**) Initial non-enhanced head computed tomography (CT) 3.3 h after stroke onset shows no intracranial hemorrhage. (**B**) CT angiography of the head and neck immediately after intravenous thrombolytic treatment shows no significant stenosis or occlusion of the carotid and major intracranial arteries except bilateral mild to moderate atherosclerosis of carotid and fenestration of the left proximal middle cerebral artery

A 52-year-old man arrived at the emergency department with a chief complaint of right-sided weakness since 3 h. He had a history of hypertension. Neurological examination revealed right-sided hemiparesis (manual muscle test grade of 3 in the right arm and 4 in the right leg; grade 0 meaning no strength, grade 5 full strength) and hypoesthesia. The National Institutes of Health Stroke Scale score was 4. His blood pressure was 166/109 mm Hg. To avoid delaying thrombolysis, we first chose non-contrast CT to exclude intracranial hemorrhage. Initial head CT revealed no intracranial hemorrhage and no early ischemic changes and an old lacunar cerebral infarction in the left putamen (Fig. 1A). The prothrombin time/international normalized ratio and the activated partial thromboplastin time were normal. Intravenous treatment with tenecteplase of 16 mg (0.25 mg/kg) was started 3.8 h after stroke onset. CTA of the head and neck was performed using 50 ml of intravenous iopamidol-370 and flow rate of 4.0 ml/s 0.5 h after IVT. The CTA showed bilateral mild to moderate atherosclerosis of the carotid and fenestration of the left proximal middle cerebral artery without any hemodynamically significant stenosis or occlusion of the carotid and major intracranial arteries (Fig. 1B). Then, he was transferred to the stroke unit for further evaluation.

**Fig. 2:**
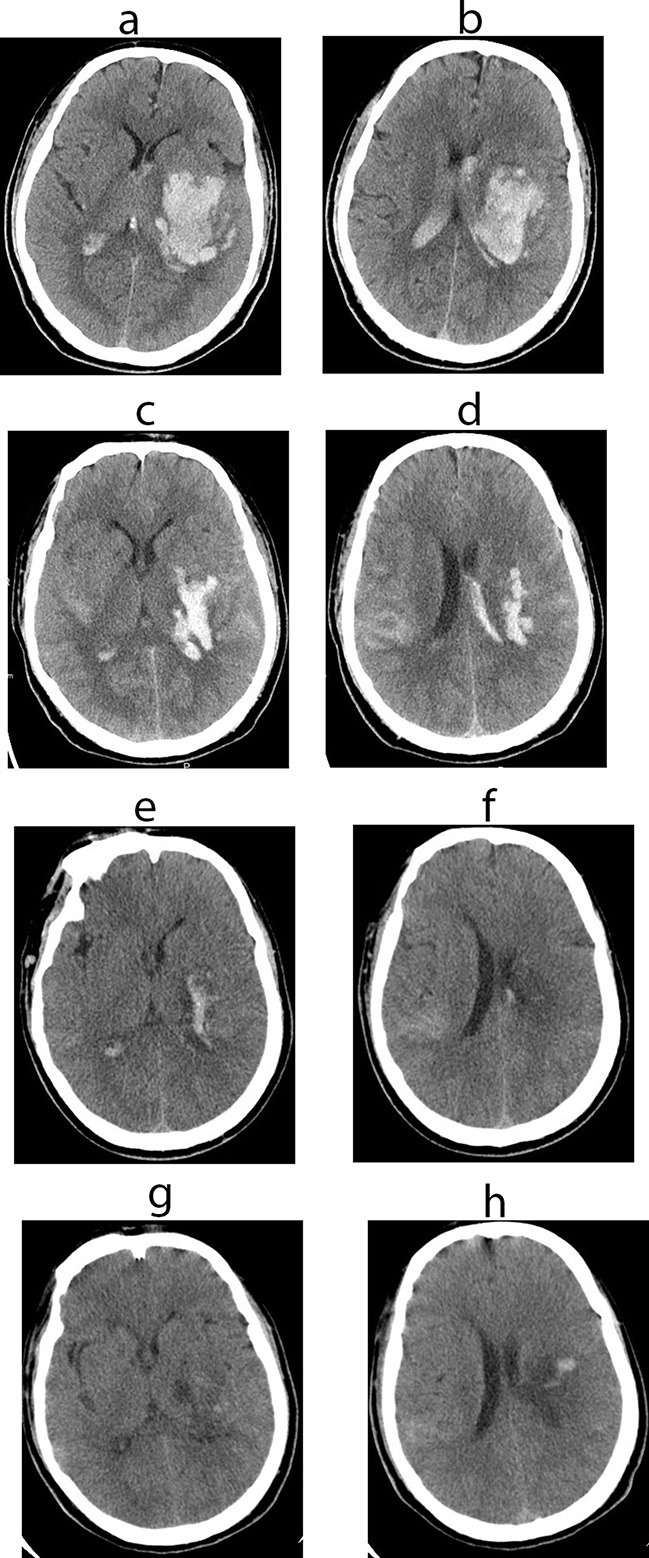
(**A** and **B**) Immediate head CT after deterioration of neurological symptoms shows hyperdense lesions with mild space-occupying effect in the left basal ganglia and both lateral ventricles. (**C** and **D**) Follow-up CT after 5 h shows significant resolution of hyperdense lesions and and hyperdensity of the right upper insula. (**E** and **F**) Two days later, CT shows almost complete resolution of hyperdense lesions in the lateral ventricles and a small of amount residue in the basal ganglia and insular, without obvious surrounding edema and mass effect. (**G** and **H**). At eight days after intravenous thrombolytic treatment, CT shows only some scattered small petechiae in the left infarcted area

At about 1.5 h after CTA, the right-sided hemiparesis deteriorated with a manual muscle test grade of 1 in the right arm and 2 in the right leg, accompanied by drowsiness, aphasia, and urinary incontinence. Immediate head CT showed hyperdense lesions with mild space-occupying effect in the left basal ganglia and both lateral ventricles, which.

had a mean Hounsfield unit (HU) of 55 (range from 24 HU to 79 HU) (Fig. 2A and B). He was transferred to our department for further treatment. The follow-up CT after 5 h showed that the hyperdensity in the left basal ganglia and lateral ventricles was reduced in size. A mild hyperdensity of the right posterior insular cortex was found (Fig. 2C and D). Two days later, CT showed that the hyperdense lesions in the lateral ventricles had almost completely disappeared and only a small amount remained in the basal ganglia and insular, without obvious surrounding edema and mass effect (Fig. 2E and F). The hyperdensity in the right posterior insular cortex remained, probably representing a mild HT (Fig. 2F). Only some scattered small petechiae could be seen in the left infarcted area on CT eight days after intravenous thrombolytic treatment (Fig. 2G and H). The patient was improving with physical, occupational and speech therapies during hospitalization and was eventually discharged to a rehabilitation center.

## Discussion and conclusions

Hyperdense lesions on non-enhanced CT are frequently seen after diagnostic or interventional cerebral angiography in patients with AIS, which represents contrast extravasation and/or HT. Disruption of the blood-brain-barrier (BBB) is associated with contrast extravasation and HT in AIS patients. The pathophysiology of disruption of the BBB in this population involves ischemic/reperfusion injury, contrast neurotoxicity, toxicity secondary to thrombolytic agents, and procedure-related direct vessel damage [3, 4]. Limited injury to the BBB can lead to contrast media extravasation without blood; however, with the progression of BBB disruption, the extravasation of large cellular blood elements takes place [4].

Contrast extravasation can be differentiated from HT using head magnetic resonance imaging (MRI) in patients with AIS. The MR signal of HT depends on the sequence used varies with time after hemorrhage onset. Contrast extravasation demonstrates an iso- or hyperintensity signal compared with that of the normal gray matter on B0-DWI (diffusion weighted MRI), T2WI, and gradient recalled-echo [5]. Recently, dual-energy CT (DECT) has been proven to have excellent accuracy in differentiating ICH from contrast extravasation after endovascular thrombectomy for AIS [6]. DECT uses attenuation measurements acquired with two selected energy spectra (e.g., 80 kV and 140 kV) to differentiate contrast from hemorrhage rapidly [7]. Thus, the early use of brain DECT or MRI is recommended to distinguish between ICH and contrast extravasation after AIS. However, the availability of these scanners may be limited in daily clinical practice.

Although contrast extravasation mimicking ICH is commonly seen on follow-up radiological imaging after endovascular treatment, this phenomenon has been rarely reported after intravenous thrombolytic treatment in patients with AIS. To our knowledge, only Park et al. [8] and Khalily et al. [9], respectively, reported a case of contrast extravasation mimicking intraventricular hemorrhage and a case of contrast extravasation mimicking lobe hemorrhage following intravenous thrombolytic treatment. In our case, the hyperdensity was located in the left basal ganglia and lateral ventricles with a large volume, which is more likely considered as ICH with intraventricular extension. Unfortunately, due to the lack of DECT availability and the situation of our patient, he did not obtain DECT or MRI examination. However, based on serial follow-up head CT, we were inclined to diagnose the intracranial hyperdense lesion as contrast extravasation combined with symptomatic ICH in our case. First, contrast agents generally wash out within 24–48 h [10, 11], while ICH persists for days to weeks. In our case, the majority of intracranial hypertense lesions rapidly disappeared on the serial follow-up CT within 24 h, suggesting that contrast extravasation would be the main cause. Second, despite the large size of the intracranial hyperdense lesion, there was no obvious mass effect, which is not consistent with the radiological features of massive ICHs. Third, the absence of significant edema surrounding the hyperdense lesion in the brain parenchyma provided another evidence for the possible existence of contrast [12]. Additionally, previous studies have demonstrated that a hyperdense lesion with a maximal HU > 90 could be defined as contrast extravasation on a 24-hour follow-up CT [3, 10], and an average attenuation < 50 HU in the most visually hyperattenuating hyperdense lesion had 100% specificity for identification of contrast extravasations in AIS patients after endovascular treatment [12]. Therefore, the contrast mixture with blood may explain why the maximal HU and mean HU of the hyperdensity were 79 and 55 in our case, respectively. Finally, the neurological deterioration and the residual hyperdensity in the area of infarction after 48 h may be the evidence for symptomatic ICH [13].

There are a few limitations in this report. We made the diagnosis of ischemic stroke based on the clinical presentation and a negative non-contrast CT, and could not assess stroke etiology. Therefore, CTA or MR-angiography with DWI before thrombolytic treatment should be obtained to determine the diagnosis and etiology of AIS without delaying thrombolysis. Another limitation is lack of a DECT or MRI to clarify the properties of the intracranial hyperdensity. Despite these limitations, this case is rare and deserves the attention of physicians.

In conclusion, contrast extravasation into the brain tissue and lateral ventricles is a rare event, but our case shows that it could occur after intravenous thrombolytic treatment and CTA in a patient with AIS. Most importantly, it could be misdiagnosed as ICH with intraventricular extension on non-enhanced CT, which might result in the wrong treatment like ventricular drainage. Rapid resolution of intracranial hyperdense lesions without mass effect is the key to the diagnosis of contrast extravasation on serial non-contrast CT that requires time however.

## Data Availability

All data generated or analyzed during this study are included in this published. article [and its supplementary information files].

## Abbreviations

CT: Computed tomography
AIS: Acute ischemic stroke
ICH: Intracerebral hemorrhage
CTA: Computed tomography angiography
IVT: Intravenous treatment
HT: Hemorrhagic transformation
HU: Hounsfield unit
BBB: Blood-brain-barrier
MRI: Magnetic resonance imaging
DWI: Diffusion weighted MRI
DECT: Dual-energy CT
